# Renin-Angiotensin-System Inhibitors Are Associated With Lower In-hospital Mortality in COVID-19 Patients Aged 80 and Older

**DOI:** 10.3389/fcvm.2022.916509

**Published:** 2022-06-17

**Authors:** Francesco Spannella, Federico Giulietti, Chiara Di Pentima, Massimiliano Allevi, Valentina Bordoni, Andrea Filipponi, Sara Falzetti, Caterina Garbuglia, Samuele Scorcella, Piero Giordano, Riccardo Sarzani

**Affiliations:** ^1^Internal Medicine and Geriatrics, IRCCS INRCA, Ancona, Italy; ^2^Department of Clinical and Molecular Sciences, University “Politecnica delle Marche”, Ancona, Italy

**Keywords:** COVID-19, renin-angiotensin-system, older adults, mortality, propensity score matching

## Abstract

**Background:**

Older adults are at higher risk of morbidity and mortality for coronavirus disease 2019 (COVID-19). Renin-angiotensin-system inhibitors (RASi) were found to have a neutral or protective effect against mortality in COVID-19 adult patients.

**Aims:**

We investigated whether this association was confirmed also in COVID-19 older patients.

**Methods:**

This is a prospective observational study on 337 hospitalized older adults (aged 80 years and older). We classified the study population according to usage of RASi before and during hospitalization. A propensity score analysis was also performed to confirm the findings.

**Results:**

The mean age was 87.4 ± 6.1 years. Patients taking RASi at home were 147 (43.6%). During hospitalization, 38 patients (11.3% of the entire study population) discontinued RASi, while 57 patients (16.9% of the entire study population) started RASi. In-hospital mortality was 43.9%. Patients taking RASi during hospitalization (patients who maintained their home RASi therapy + patients who started RASi during hospitalization) had a significantly lower in-hospital mortality than untreated patients [HR 0.48 (95% CI: 0.34–0.67)], even after adjustment for required respiratory support, functional status, albumin, inflammation, and cardiac biomarkers. The analysis of the groups derived from the “propensity score matching” (58 patients in each group) confirmed these results [HR 0.46 (95% CI: 0.23–0.91)].

**Discussion:**

Despite the high risk of death in older COVID-19 patients, RASi therapy during hospitalization was associated with a clinically relevant lower in-hospital mortality, likely due to the benefit of RAS modulation on the cardiopulmonary system during the acute phase of the disease.

**Conclusion:**

Our findings confirm the protective role of RASi even in COVID-19 patients aged 80 years and older.

## Introduction

Age and multimorbidity are the main risk factors for death in patients affected by coronavirus disease 2019 (COVID-19) (1). In addition to older age, cardiovascular diseases (CVD), chronic obstructive pulmonary disease (COPD), chronic kidney disease (CKD), diabetes, and obesity are the comorbidities most associated with higher in-hospital mortality (2, 3). In this context, CVD, mainly related to arterial hypertension and/or diabetes mellitus, play a key role in the prognosis of COVID-19, given its prevalence worldwide, that increases with increasing age (4, 5). At the same time, younger age and the absence of both CKD and heart failure (HF) were found to be associated with better COVID-19 outcomes (6). Angiotensin-converting-enzyme inhibitors (ACEi) and angiotensin-receptor-blockers (ARB) are cornerstone drugs for CVD by lowering blood pressure (BP) and exerting anti-hypertrophic, anti-fibrotic, and anti-inflammatory activities, leading to reduced progression of heart, renal and vascular damage, and lower CV morbidity and mortality (7). Therefore, many older subjects are currently taking, or should take as per clinical indication, renin-angiotensin-system inhibitors (RASi), given the high prevalence of hypertension and hypertension-related CVD in this population.

After initial unfounded speculations questioning the safety of RASi in COVID-19, extensive literature has been published on this topic, disproving those initial fears and confirming either neutral or even protective effects of RASi on severity and mortality for COVID-19 (8). However, the clinical evidence in the older population, which is both the one most treated with RASi and the one most at risk for COVID-19 morbidity and mortality, is still scarce (8, 9). Therefore, the aim of our study was to evaluate the association between RASi therapy and in-hospital mortality in patients aged 80 years and older admitted for COVID-19.

## Materials and Methods

### Study Design and Population

We performed a prospective observational study on 337 older adults, consecutively admitted from March 2020 to March 2021 to the COVID-19 wards of the Italian National Institute of Health and Science on Aging (INRCA: Istituto Nazionale di Riposo e Cura per Anziani), which is the only organization specifically focused on geriatric care and gerontological research in Italy. Our hospital is dedicated to scientific research and the care of older subjects (mostly aged 80 years and older), which are usually excluded from clinical trials and in which scientific evidence is still scarce. We considered the following inclusion criteria: age ≥80 years, access from the Emergency Department, and confirmed COVID-19. The diagnosis of COVID-19 was made on the basis of compatible clinical characteristics (at least one symptom/sign, such as fever, cough, dyspnea or tachypnea, peripheral oxygen desaturation, headache, diffuse arthralgia, and/or myalgia) and confirmed by the virus detection by reverse transcriptase-polymerase chain reaction (RT-PCR) from a nasal/oro-pharyngeal swab on admission. We excluded patients having conditions with a life expectancy of <1 year or terminally ill patients (end-stage renal disease or dialysis, decompensated cirrhosis, advanced cancer, severe dementia, or bed rest syndrome) and patients managed with a palliative care approach upon admission.

### Clinical Parameters

Medical history and laboratory parameters were collected on each enrolled patient on admission. We considered the following laboratory parameters: hemoglobin (Hgb), white blood cells (WBC) count, neutrophil and lymphocyte count, estimated glomerular filtration rate (eGFR), N-terminal pro B-type natriuretic peptide (NT-proBNP), and albumin. Moreover, we also considered the most common laboratory parameters that were found to be associated with COVID-19 severity in previous studies (10): C-reactive protein (CRP), high-sensitive cardiac troponin T (hs-cTnT), D-dimer, serum ferritin, interleukin-6 (IL-6), lactate dehydrogenase (LDH), liver enzymes, and PaO2/FiO2 ratio (P/F, the ratio of arterial oxygen partial pressure in mmHg to fractional inspired oxygen expressed as a fraction, not a percentage) at arterial blood gas analysis. The GFR was estimated using the CKD-EPI equation. The age-adjusted NT-proBNP cutoff of 1,800 pg/ml, proposed by Januzzi et al. (5) was used to diagnose acute decompensated HF. The need for respiratory support during hospitalization was classified as follows: no need for oxygen therapy, need for oxygen therapy, and need for ventilatory support. SARS-CoV-2-related pneumonia was defined as the presence of confirmed SARS-CoV-2 infection and at least one new radiological finding of pneumonia (i.e., ground glass opacities, crazy-paving pattern, lobular, and sub-segmental areas of consolidation) on chest X-ray or chest high-resolution computed tomography (HRCT) during hospitalization. Patients were defined as affected by arterial hypertension in the presence of a documented history of hypertension and/or if they were treated with at least one anti-hypertensive drug at home. On admission, BP was measured with an automatic oscillometric BP device (Omron Healthcare, Japan), using the correct cuff sizes according to arm circumference with the patient's arm kept at heart level during the measurement. The history of coronary artery disease (CAD), heart failure (HF), atrial fibrillation (AF), previous stroke/transient ischemic attack (TIA), and chronic obstructive pulmonary disease (COPD) was defined according to the patient's medical history or previous patient's medical reports. Type 2 diabetes mellitus was defined according to international guidelines or treatment with at least one anti-diabetic drug at home (11). In addition to RASi therapy, the following CV drug classes were also reported in order to better characterize the study population: beta-blockers, calcium antagonists, diuretics, mineralocorticoid receptor antagonists, statins, antiplatelets, and anticoagulants.

### Geriatric Comprehensive Assessment

As previously reported (12), to evaluate patients' functional status, the 7-point MDS Activities of Daily Living (ADL) Hierarchy Scale was used. The ADL Hierarchy Scale groups activities of daily living according to the stage of the disablement process in which they occur (13). The ADL Hierarchy Scale ranges from 0 (no dependence) to 6 (total dependence). ADL disability was categorized as follows: no impairment (ADL Hierarchy Scale score < 2), assistance required (ADL Hierarchy Scale score 2–4), and dependence (ADL Hierarchy Scale score ≥ 5). Cognitive impairment was based on a previous documented diagnosis, given that the result of any cognitive test could have been altered by the acute phase. The Geriatric Index of Comorbidity (GIC) was used to determine the burden of comorbidities, and it was categorized as low comorbidity (GIC classes 1 or 2) and high comorbidity (GIC classes 3 or 4) (14).

### Statistical Analysis

Data were analyzed using the Statistical Package for Social Science version 21 (SPSS Inc., Chicago, Illinois, USA). A value of *p* < 0.05 was defined as statistically significant. Continuous variables were checked for normality and expressed as mean ± standard deviation or median and interquartile range for significantly skewed variables. Categorical variables were expressed in percentages. The χ^2^ test was used to analyze the differences between categorical variables. Unpaired two-tailed *t*-test (Student's *t*-test) was used to compare quantitative variables normally distributed, and Mann–Whitney U test was used to compare quantitative variables not normally distributed. Kaplan–Meier curves were drawn for mortality according to RASi use and compared with the log-rank test. Cox regression models were built with in-hospital mortality as the dependent variable and the use of RASi as the independent variable adjusted for several confounders. Log-minus-log plots were used to assess proportional hazards assumptions. Variables with non-normal distributions have been transformed into natural logarithms to normalize their distributions and therefore to be inserted into Cox regression models. We considered in-hospital mortality as an outcome. For this scope, we performed the main analysis comparing two groups: patients taking RASi during hospitalization, which included patients who maintained their home RASi therapy + patients who started RASi during hospitalization, and patients not taking RASi during hospitalization, that included patients who had never taken RASi + patients who discontinued RASi during hospitalization. Home RASi therapy was maintained unless contraindicated due to the increased risk of serious adverse events, while the decision to start or discontinue RASi during hospitalization was determined by clinical indication according to guidelines (15–17) and principles of “good clinical practice” (i.e., according to BP values, serum potassium levels, renal function, and the presence of HF). In a sensitivity analysis, we also classified the study population into four groups according to RASi therapy and evaluated their associations with mortality: patients who had never taken RASi, patients who discontinued RASi during hospitalization (within the first 48 h of admission), patients who started RASi during hospitalization, and patients who maintained their home RASi therapy (Supplementary Figure 1). The Bonferroni *post-hoc* test was used to compare the different RASi subgroups. The main variables associated with mortality were used as covariates in the adjusted models, including required respiratory support, functional status, albumin, inflammation, and cardiac biomarkers. Adjusted models were also built, taking into account the number of events per variable (EPV), in order to avoid misleading findings (18, 19).

Subsequently, to assess the relationship between RASi therapy and in-hospital mortality, patients were stratified into two equally sized groups based on whether they are taking RASi therapy during hospitalization or not. A propensity score was obtained by means of multiple logistic regression. In this analysis, we excluded subjects who discontinued the drug during the hospital stay in order to limit confounding by contraindication. We included the following variables in the score: age, sex, history of HF, history of atrial fibrillation (AF), previous stroke/transient ischemic attack (TIA), cognitive impairment, systolic blood pressure (BP) on admission, WBC, neutrophil and lymphocyte count, albumin, eGFR, NT-proBNP, hs-cTnT, CRP, D-dimer, serum ferritin, need for respiratory support, P/F, ADL Hierarchy Scale, and GIC. Matching was then performed on the log-transformed propensity score in a 1:1 fashion with a caliper of 0.05 in order to account for the different baseline characteristics between the RASi and no-RASi groups (controls).

## Results

### General Characteristics and In-hospital Mortality

General characteristics of the study population are described in Table 1. The mean age was 87.4 ± 6.1 years, with a female prevalence of 55.8%. The main length of stay was 14.0 ± 10.6 days. The more prevalent comorbidities were hypertension and cognitive impairment, followed by the history of HF and AF. SARS-CoV-2-related pneumonia was found in 88% of the study population. More than half of patients (51.4%) needed oxygen therapy, and 37.7% of patients needed ventilatory support during hospitalization. Acute decompensated HF, defined by NT-proBNP levels ≥1,800 pg/ml, was found in 46.6% of patients. The prevalence of CV drugs other than RASi taken at home by the study population was the following: 36.8% for beta-blockers, 23.8% for calcium antagonists, 50.2% for diuretics, 13.2% for mineralocorticoid receptor antagonists, 32.3% for statins, 36.4% for antiplatelets, and 31.0% for anticoagulants.

**Table 1:** General characteristics of the entire study population and according to RASi therapy during hospitalization.

**Clinical parameters**	**Study population (n°337)**	**No RASi during hospitalization (n° 171)**	**RASi during hospitalization (n° 166)**	***p*-value**
Age (years)	87.4 ± 6.1	88.1 ± 6.6	86.6 ± 5.5	**0.032**
Sex (Female)	55.8%	52.6%	59.0%	0.237
BMI (kg/m^2^)	25.2 ± 4.0	24.4 ± 3.8	26.0 ± 4.1	**0.003**
ADL Hierarchy scale: Assistance required	26.7%	19.6%	33.6%	**<0.001**
ADL Hierarchy scale: Dependence	47.0%	61.6%	32.9%	
GIC (high comorbidity)	74.1%	76.6%	71.6%	0.340
History of hypertension	75.8%	64.2%	87.7%	**<0.001**
History of CAD	21.7%	19.9%	23.5%	0.421
History of HF	32.8%	32.1%	33.5%	0.783
Type II diabetes mellitus	22.6%	19.9%	25.3%	0.234
History of AF	27.9%	26.3%	29.5%	0.512
Previous stroke/TIA	16.3%	17.5%	15.1%	0.537
History of COPD	21.0%	19.3%	22.7%	0.483
Cognitive impairment	65.0%	72.9%	56.6%	**0.004**
Need for oxygen therapy	51.4%	52.7%	39.5%	0.173
Need for ventilatory support	37.7%	50.0%	35.8%	
Systolic BP (mmHg)	132.3 ± 20.5	127.4 ± 20.0	137.2 ± 19.9	**<0.001**
Diastolic BP (mmHg)	73.7 ± 12.4	72.4 ± 12.2	74.9 ± 12.4	0.082
**Laboratory parameters**
Hgb (g/dl)	12.6 ± 1.7	12.5 ± 1.9	12.6 ± 1.6	0.825
WBC (n/mm^3^)^*^	7,185 (5,170–11,167)	7,950 (5,210–12,180)	6,800 (5,130–10,120)	0.058
Neutrophils (n/mm^3^)^*^	5,745 (3,533–9,253)	5,930 (3,590–10,380)	5,370 (3,515–8,040)	0.120
Lymphocytes (n/mm^3^)^*^	885 (638–1,330)	910 (635–1,495)	880 (630–1,290)	0.554
eGFR (ml/min/1.73 m^2^)	51.6 ± 24.6	49.7 ± 26.4	53.4 ± 22.5	0.151
Albumin (g/dl)	3.3 ± 0.5	3.2 ± 0.5	3.4 ± 0.4	**<0.001**
AST (U/L)^*^	29 (21–44)	30 (20–48)	27 (21–42)	0.252
ALT (U/L)^*^	18 (12–30)	18 (12–31)	18 (12–29)	0.941
D-dimer (μg/ml)^*^	1,500 (770–2,940)	1,400 (835–3,770)	1,555 (738–2,618)	0.358
LDH (U/L)^*^	313 (235–482)	319 (242–550)	298 (226–445)	0.067
Serum ferritin (ng/ml)^*^	517 (228–963)	558 (253–1,072)	483 (225–845)	0.081
NT-proBNP (pg/ml)^*^	1,581 (624–4,953)	1,579 (644–4,927)	1,620 (599–4,985)	0.956
hs-cTnT (ng/L)^*^	38.8 (23.0–89.1)	44.1 (25.5–94.2)	35.0 (21.9–73.0)	0.066
CRP (mg/dl)^*^	6.05 (2.11–12.13)	7.67 (3.09–15.99)	4.93 (1.62–10.57)	**0.005**
IL-6 (pg/ml)^*^	43.0 (23.4–101.4)	43.0 (19.3–110.3)	45.8 (24.0–88.8)	0.968
P/F	266.5 ± 95.4	261.6 ± 97.9	273.2 ± 92.1	0.461

* *The Mann–Whitney U test was used for the comparison between the two groups (no RASi during hospitalization vs. RASi during hospitalization). Where not specified, unpaired two-tailed t-test was used for comparison of quantitative variables and χ^2^ test was used for comparison of categorical variables. Bold indicates significance. RASi, renin-angiotensin-system inhibitors; BMI, body mass index; GIC, geriatric index of comorbidity; CAD, coronary artery disease; HF, heart failure; AF, atrial fibrillation; TIA, transient ischemic attack; COPD, chronic obstructive pulmonary disease; BP, blood pressure; Hgb, hemoglobin; WBC, white blood cells; eGFR, estimated glomerular filtration rate; AST, aspartate aminotransferase; ALT, alanine aminotransferase; LDH, lactate dehydrogenase; NT-proBNP, N-terminal pro B-type natriuretic peptide; hs-cTnT, high-sensitive cardiac troponin T; CRP, C-reactive protein; IL-6, interleukin-6; P/F, PaO2/FiO2 ratio (the ratio of arterial oxygen partial pressure in mmHg to fractional inspired oxygen expressed as a fraction, not a percentage)*.

A total of 148 patients (43.9%) died during hospitalization. Mortality was higher in patients who required ventilatory support compared to patients on oxygen therapy alone (61.3 vs. 40.8%, *p* < 0.001). Deceased patients were older, mostly male, with a higher prevalence of HF, AF, and previous stroke/TIA (Supplementary Table 1). Moreover, they had a greater burden of comorbidities and a higher prevalence of dependence in ADL. Higher levels of both inflammation and cardiac biomarkers, as well as lower BP on admission, eGFR, and albumin, were also associated with the increased mortality (Supplementary Table 1).

### RAS Inhibitor Therapy and In-hospital Mortality

Patients already taking RASi at home were 147 (43.6%). During hospitalization, 38 patients discontinued RASi (11.3% of the entire study population), whereas 57 patients (16.9% of the entire study population) started RASi during hospitalization (Supplementary Figure 1). The main reasons for RASi discontinuation (alone or in combination) were the following: momentary inability to take medications orally (57.9%), hypotension (15.8%), and increase of creatinine level ± hyperkalemia on admission (31.6%), while the main reasons for RASi initiation were the following: HF (77.6%) and/or high BP (28.1%) on admission.

**Figure 1:**
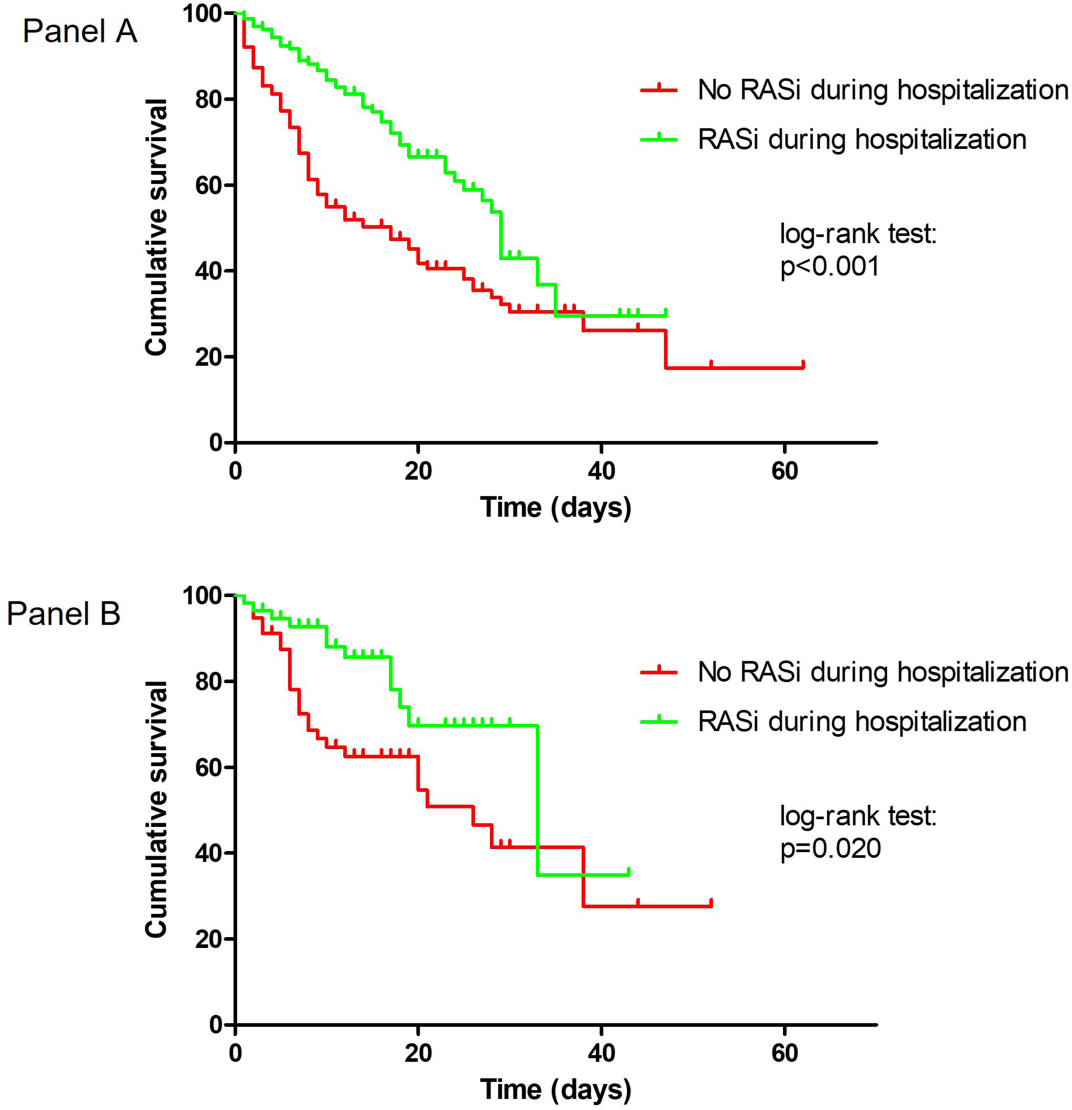
Kaplan–Meier survival curves according to RASi therapy during hospitalization. **(A)** Overall population. **(B)** Propensity score matched groups.

There was no association between home RASi therapy and in-hospital mortality [40.1 vs. 46.8%; HR 0.79 (95% CI: 0.56–1.10), *p* = 0.156], while patients taking RASi during hospitalization (patients who maintained their home RASi therapy + patients who started RASi during hospitalization) had lower in-hospital mortality than patients not taking RASi during hospitalization (patients who had never taken RASi + patients who discontinued RASi during hospitalization) [30.1 vs. 57.3%; HR 0.48 (95% CI: 0.34–0.67), *p* < 0.001] (Figure 1A).

The general characteristics of patients taking RASi during hospitalization (49.2% of the entire study population) are described in Table 1. They were younger with higher body mass index (BMI), higher prevalence of hypertension, higher BP on admission, higher albumin levels, lower prevalence of both cognitive impairment and dependence in ADL, and also lower CRP levels than patients not taking RASi. No difference in the prevalence of RASi therapy during hospitalization was found between patients who needed ventilatory support and patients who needed oxygen therapy alone.

The association between RASi during hospitalization and in-hospital mortality remained significant even after adjustment for several possible confounders, including required respiratory support, functional status, albumin, inflammation, and cardiac biomarkers (Table 2). Among the deceased patients, 94.4% had SARS-CoV-2-related pneumonia. Regarding pneumonia-related mortality, a lower mortality rate was observed in patients treated with RASi compared with untreated patients [34.8 vs. 59.1%; HR 0.54 (95% CI: 0.38–0.76), *p* = 0.001] (refer to Supplementary Table 2 for adjusted analyses).

**Table 2:** Cox regression analyses for in-hospital mortality according to RASi therapy during hospitalization.

	**HR**	**95% CI**	***P*-value**
Model 1	0.52	0.37–0.73	<0.001
Model 2	0.54	0.38–0.77	0.001
Model 3	0.60	0.36–0.99	0.049
Model 4	0.57	0.33–0.97	0.038

*CI, confidence interval. Model 1: adjusted for age and sex. Model 2: adjusted for age, sex, and respiratory support. Model 3: adjusted for age, sex, systolic blood pressure, ADL Hierarchy Scale, Ln (D-dimer), Ln (serum ferritin), and Ln (high-sensitive cardiac troponin T). Model 4: adjusted for age, sex, cognitive impairment, albumin, estimated glomerular filtration rate, Ln (C-reactive protein), and Ln (NT-proBNP)*.

**Figure 2:**
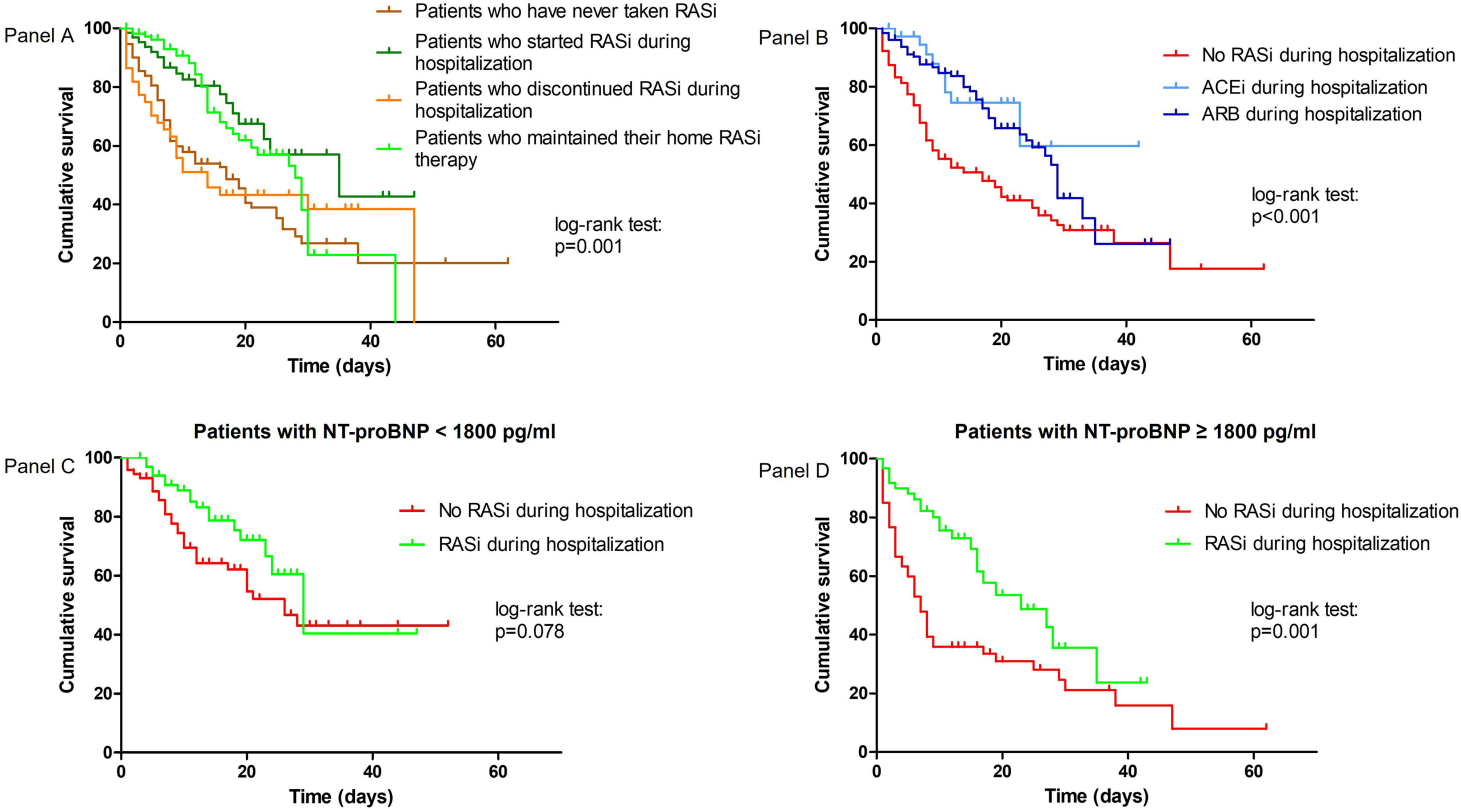
Kaplan–Meier survival curves according to change in RASi therapy during hospitalization **(A)**, according to ACEi or ARB therapy during hospitalization **(B)**, and according to RASi therapy during hospitalization and admission NT-proBNP levels **(C,D)**.

In the sensitivity analysis (Figure 2A), patients who started RASi during hospitalization had lower mortality than patients who had never taken RASi [26.3 vs. 55.6%; HR 0.39 (95% CI: 0.23–0.69), *p* = 0.001]. The same protective association was found in patients treated both at home and during hospitalization compared to patients who had never taken RASi [32.1 vs. 55.6%; HR 0.51 (95% CI: 0.34–0.76), *p* = 0.001]. Patients who maintained their home RASi therapy showed a lower mortality compared to patients who discontinued RASi during hospitalization [32.1 vs. 63.2%; HR 0.57 (95% CI: 0.33–0.99), *p* = 0.049]. These findings were confirmed even after adjustment for covariates, although statistical significance was lost for the latter association (refer to Supplementary Table 3 for adjusted analyses).

Among patients taking RASi during hospitalization, 38 patients (22.9%) took an ACEi, with the highest prevalence for perindopril (68.0%), while 128 patients (77.1%) took an ARB, with the highest prevalence for losartan (38.8%) and candesartan (37.2%). Patients treated with either ACEi [HR 0.42 (95% CI: 0.21–0.83), *p* = 0.013] or ARB [HR 0.49 (95% CI: 0.34–0.21), *p* < 0.001] showed a significant lower in-hospital mortality compared to untreated patients, while no difference on the risk of in-hospital mortality was found between ACEi and ARB groups [23.7 vs. 32.0%, HR 1.08 (95% CI: 0.52–2.21), *p* = 0.861] (Figure 2B).

The protective role of RASi therapy during hospitalization was likely more relevant in patients with NT-proBNP ≥ 1,800 pg/ml [in-hospital mortality rate: 36.5% for RASi users vs. 75.8% for RASi non-users, HR 0.45 (95% CI: 0.27–0.75), *p* = 0.002] than in patients with NT-proBNP < 1,800 pg/ml, in which statistical significance was lost [in-hospital mortality rate: 25.0% for RASi users vs. 44.0% for RASi non-users, HR 0.57 (95% CI: 0.32–1.03), *p* = 0.066] (Figures 2C,D; Supplementary Table 4 for adjusted analyses).

### Propensity Score Matched Groups

After excluding subjects who discontinued RASi therapy during hospitalization, 58 patients on RASi therapy during hospitalization were matched with as many untreated patients. Except for the history of hypertension, which was more prevalent in the RASi group, the two groups had similar general characteristics (Supplementary Table 5). The main findings were confirmed even in the propensity score matched groups. A lower mortality rate was observed in patients taking RASi compared with patients not taking RASi during hospitalization [20.7 vs. 44.8%, HR 0.46 (95% CI: 0.23–0.91), *p* = 0.025] (Figure 1B).

## Discussion

We found that taking RASi during hospitalization was associated with lower in-hospital mortality in patients aged 80 years and older admitted for COVID-19. This association was confirmed even after adjustment for several confounders, and the protective effect was most evident in patients with acute decompensated HF, based on NT-proBNP admission levels.

The classic risk factors for CVD, such as hypertension, diabetes, and CKD, are also risk factors for severe COVID-19 and death (2, 20). A large amount of data suggest that RASi, cornerstone drugs for CVD, are likely to affect COVID-19 course, though not agreed by all studies (21, 22). Among the more recent studies, two nationwide cohort studies conducted in France and Sweden reported that taking RASi was associated with a lower risk of COVID-19 hospitalization and death (23, 24). An Italian nationwide observational study found that ACEi/ARB did not affect the risk of more severe COVID-19 (2, 6). In the HOPE-COVID-19 international investigator-initiated registry, patients receiving RASi had better outcomes, including rates of respiratory failure, need for mechanical ventilation or prone positioning, sepsis, and renal failure (25). Several meta-analyses have been conducted to summarize the available evidence on this topic. A large meta-analysis enrolling 101949 COVID-19 patients found a significant association between treatment with RASi and mortality reduction among COVID-19 patients with hypertension (26). In hypertensive patients, a lower risk of ventilatory support was also found in the ACEi/ARB group (27). Other meta-analyses on 31 cohort studies and 86 studies found no significant association between RASi therapy and risk of infection and adverse outcome for COVID-19, indicating on the safety of these drugs and the need not to discontinue them, without clinical indication (8, 28).

Despite this large number of studies on this topic, only a few studies have been performed so far to investigate what happens at an older age (9), such as that of our study population (mean age: 87.4 ± 6.1 years). A French observational study, conducted in a geriatric department, showed a lower mortality rate in older patients (mean age: 86.3 ± 8.0 years) taking ACEi/ARB compared with patients not taking these drug classes (29). Their population was similar to ours in clinical characteristics. In agreement with our results, the association found was independent from indicators of disability and disease severity (29). Oliveira et al. (30) found a 73% lower risk of mortality for patients chronically taking RASi in a geriatric population with an average age slightly lower than ours (mean age: 80.9 ± 8.7 years). Moreover, a recently published Italian study found that antecedent use of RASi was associated with lower mortality in hypertensive COVID-19 patients aged 68 years and older (31). In our sample, we found a lower risk of at least 50% of in-hospital mortality in patients treated with RASi during hospitalization, regardless of disease severity and underlying medical conditions. We found no significant prognostic differences between ACEi and ARB, although the majority of our patients took an ARB (77.1%). This high prevalence was likely related to the greater preference to use ARBs in our setting of comorbid older adults admitted for an acute respiratory illness, given their fewer side effects (especially dry cough, a well-known possible side effect of ACEi, but also a prevalent symptom of COVID-19). Our data on older patients confirm the results of the other observational studies on hospitalized geriatric COVID-19 patients, providing additional evidence on the benefit of RASi use in this peculiar population. All these findings are also consistent with our previous studies. Indeed, in the pre-COVID era, we found that ACEi/ARB therapy was associated with a lower risk of death, likely due to better CV protection and a higher CV resilience to acute illness in older patients hospitalized for several medical conditions, including pneumonia and other infections (12).

On the contrary, we found a likely higher risk of death in COVID-19 patients who discontinued RASi during hospitalization in our study. Our findings are in line with most previous studies on younger populations that showed how the discontinuation of CV therapy, such as RASi and beta-blockers, was associated with an increased risk of intensive care unit admission and death from SARS-CoV-2 infection, independent of the admission disease severity (32–34). ACEi/ARB withdrawal was found to lead to a greater risk of complications and mortality in hospitalized COVID-19 patients that were previously taking these drugs as per indication (35). On the contrary, several large systematic reviews, cohort studies, and randomized clinical trials (RCTs), mainly on younger populations, showed how their assumption/continuation was absolutely not harmful, firmly advising against their discontinuation (8, 22, 28, 36).

Several hypothetical mechanisms could explain these findings. RASi therapy strongly affects the interactions between SARS-CoV-2 and RAS arms and exerts multiple protective actions on CV target organ damage.

An imbalance between the two opposing arms of RAS (“classic RAS” and “anti-RAS”) is very likely to promote and accelerate lung injury in COVID-19, through the internalization and shedding of ACE2, following the binding of the SARS-CoV-2 surface spike protein. The downregulation of the ACE2-angiotensin (1–7)-Mas receptor pathway (“anti-RAS” arm), together with the consequent hyper-activation of the ACE-angiotensin II-angiotensin II type 1 receptor (AT1R) pathway (“classic RAS” arm), induces pulmonary vasoconstriction and microvascular damage with increased vascular permeability, resulting in an enlarged and “leaky” pulmonary microvasculature and damaged alveoli filled with plasma proteins. Consequently, there is a secondary production of inflammatory cytokines, which promotes apoptosis in alveolar epithelial cells and extracellular matrix synthesis, leading to lung fibrosis (37). Older adults, especially those with CV comorbidities, already have reduced ACE2 levels compared to younger adults (38) and an upregulated angiotensin II proinflammatory pathway. This proinflammatory background can be exacerbated by SARS-CoV-2 infection in these older patients (39). On the contrary, ACEi and ARBs can facilitate ACE2 activity through rebalancing of the “anti-RAS” arm, although a direct action of these drugs on the increase of ACE2 expression would not appear to be completely clear in humans (40–42). Indeed, treatment with ARBs may counteract the RAS imbalance through AT1R modulation, shifting the pathway toward anti-RAS activity, while treatment with ACEi can increase the availability of angiotensin (1–9) and decrease degradation of angiotensin (1–7) (37). These mechanisms are in agreement with clinical findings of better outcomes in COVID-19 patients treated with RASi, likely thanks to the rebalancing of the two opposing RAS arms, in addition to the well-known protective effects on the heart and CV system.

The burden of CV comorbidities was high in our older population, as highlighted by the prevalence of hypertension, AF, and history of HF. Indeed, nearly half of the study population had acute decompensated HF, as defined by high diagnostic NT-proBNP levels, similarly found in other age-matched populations (5). High NT-proBNP levels were found to negatively affect prognosis in COVID-19 patients (43), as well as in other acute settings involving older adults (44–46). Although these high NT-proBNP levels in the older population could be, at least in part, due to other comorbidities such as CKD, AF, and anemia, they reflect the presence of functional and structural cardiac impairment. Whenever an acute intercurrent illness increases the body's metabolic demands, the development of an underlying HF is highly probable and associated with a worse outcome, especially in older comorbid patients with limited cardiac reserve. Patients with HF have elevated plasma ACE2 levels due to increased shedding of tissue ACE2, which may predispose them to the RAS imbalance mediated by SARS-CoV-2 that further depletes ACE2-mediated protection in both the heart and lungs (47, 48). Patients with COVID-19 having impaired myocardial function as well as high levels of myocardial distress markers, including NT-proBNP, had poor outcomes (49, 50). NT-proBNP is a good indicator of cardiac overload/stress. Interestingly, the association between RASi and in-hospital mortality has been found to be more evident in patients with NT-proBNP ≥ 1,800 pg/ml in our study. In this context, RASi therapy may protect against cardiac damage, such as myocardial injury, that may occur during an acute infection in high-CV risk older patients, particularly if they already have CVD (51, 52).

### Study Limits

The large sample composed of multimorbid patients aged 80 years and older hospitalized for COVID-19 represents the main strength of our study, given the few data available on this topic regarding the geriatric population, characterized by a high risk of mortality. However, this study also has several limitations that need to be taken into account. The major limitation was the study design, which did not permit the determination of the cause-effect nature of the relations. Although we used several adjusted models and propensity score matching (secondary analyses), which is one of the best techniques to reduce the intrinsic biases of nonrandomized studies, our results should be interpreted with caution and we cannot rule out residual confounding factors. Unmeasured confounders may have persisted despite model adjustment for the main cofactors. Although the so-called healthy user-sick stopper bias cannot be totally ruled out, given the nature of the study, the confirmed results after accurate control for several confounders, including both COVID-19 severity indices and the subject's overall health, suggest the goodness of the associations found. Moreover, no difference in the prevalence of RASi use during hospitalization was found according to the need for ventilatory support or oxygen therapy alone in our population. In this study, we did not consider specific COVID-19 treatments (e.g., corticosteroids, remdesivir, and tocilizumab) because the investigation of these drugs was outside the scope of our study. Furthermore, their indications have changed during the several COVID-19 waves and a lack of homogeneity over time and among members of the medical team was therefore present given the lack of available evidence and firm recommendations on older COVID-19 patients at the time of enrollment, leading to possible biases. Finally, the main acute causes/complications that led our patients to death were not easy to be determined in our older population, where multiple comorbidities and multiple-organ failure were almost always present and autopsy was rarely performed. Generally, severe/terminal cardiorespiratory failure refractory to therapies leads these very old comorbid patients to death.

## Conclusion

Despite the overall high mortality rate, RASi therapy during hospitalization was associated with lower in-hospital mortality in COVID-19 patients aged 80 years and older, who are often excluded from clinical trials. Therefore, prospective observational studies on large older populations, like ours, are informative and able to shed new light on the management of COVID-19 in these patients. Our findings confirm the key role played by RASi even in older COVID-19 patients and the possible harm following the discontinuation of these drugs in acute conditions such as viral infections. The cardiovascular and pulmonary protective role of RAS modulation during the acute phase of COVID-19 may result in a better resilience to the acute illness and a positive impact on survival. Further *ad-hoc* studies focused on older population are needed to confirm our positive findings on the role of RASi in COVID-19, as well as in other geriatric acute settings.

## Data Availability Statement

The datasets generated and analyzed during the current study are available from the corresponding author on reasonable request.

## Ethics Statement

Clinical investigations have been conducted according to the principles expressed in the Declaration of Helsinki and its later amendments. This observational study of a “best clinical practice” experience was approved by the Local Institutional Ethics Committee (Comitato Etico INRCA). All patients (or their legal representatives) gave informed written consent to participate in this study.

## Author Contributions

RS, FS, and PG contributed to the study concept and design. VB, AF, SF, CG, and SS contributed to the acquisition of subjects and data. FS, MA, and FG contributed to the analysis and interpretation of data. FS, FG, and CD contributed to the preparation of manuscript. RS, PG, FS, and FG contributed to critical revisions of the manuscript. All authors have read and approved the submission of this manuscript.

## Funding

This research was funded by Politecnica delle Marche University (Ricerca di Ateneo to RS).

## Conflict of Interest

The authors declare that the research was conducted in the absence of any commercial or financial relationships that could be construed as a potential conflict of interest.

## Publisher's Note

All claims expressed in this article are solely those of the authors and do not necessarily represent those of their affiliated organizations, or those of the publisher, the editors and the reviewers. Any product that may be evaluated in this article, or claim that may be made by its manufacturer, is not guaranteed or endorsed by the publisher.
