# Supplementary material for: Renin-Angiotensin-System Inhibitors Are Associated With Lower In-hospital Mortality in COVID-19 Patients Aged 80 and Older
Source: Front Cardiovasc Med. 2022 Jun 17;9:916509. doi: 10.3389/fcvm.2022.916509 (PMC9247386; doi:10.3389/fcvm.2022.916509)
Supplement: Supplementary file 1 [file Data_Sheet_1.docx]

Supplementary Material

Supplementary Table S1. General characteristics of the entire study population and according to in-hospital mortality.

| *Clinical parameters* | Survivors  (n° 189) | Deceased  (n° 148) | P |
| --- | --- | --- | --- |
| Age (years) | 86.2 ± 6.1 | 88.9 ± 5.7 | **<0.001** |
| Sex (Female) | 60.8% | 49.3% | **0.035** |
| BMI (kg/m^2^) | 25.2 ± 3.4 | 25.1 ± 4.8 | 0.825 |
| ADL Hierarchy Scale: Assistance required | 31.0% | 21.1% | **<0.001** |
| ADL Hierarchy Scale: Dependence | 32.9% | 65.0% |  |
| GIC (high comorbidity) | 64.6% | 86.7% | **<0.001** |
| History of hypertension | 74.8% | 76.9% | 0.701 |
| History of CAD | 18.5% | 25.7% | 0.113 |
| History of HF | 27.5% | 39.7% | **0.020** |
| Type II Diabetes Mellitus | 22.8% | 22.3% | 0.921 |
| History of AF | 22.8% | 34.5% | **0.017** |
| Previous stroke/TIA | 12.2% | 21.6% | **0.020** |
| History of COPD | 17.1% | 26.0% | 0.068 |
| Systolic BP (mmHg) | 136.6 ± 18.7 | 127.0 ± 21.3 | **<0.001** |
| Diastolic BP (mmHg) | 75.4 ± 11.5 | 71.5 ± 13.1 | **0.009** |
| Cognitive impairment | 54.9% | 77.2% | **<0.001** |
| *Laboratory parameters* |  |  |  |
| Hgb (g/dl) | 12.5 ± 1.6 | 12.7 ± 1.9 | 0.497 |
| WBC (n/mm^3^)^*^ | 6630 (4750 – 9420) | 8800 (13300 – 5500) | **<0.001** |
| Neutrophils (n/mm^3^) ^*^ | 5030 (3320 – 7785) | 6780 (3710 – 10920) | **0.001** |
| Lymphocytes (n/mm^3^) ^*^ | 890 (610 – 1215) | 880 (655 – 1540) | 0.391 |
| eGFR (ml/min/1.73m^2^) | 57.21 ± 23.12 | 44.30 ± 24.6 | **<0.001** |
| Albumin (g/dl) | 3.4 ± 0.4 | 3.1 ± 0.5 | **<0.001** |
| AST (U/L) ^*^ | 27 (21 – 39) | 32 (21 – 51) | **0.011** |
| ALT (U/L) ^*^ | 17 (11 – 28) | 19 (12 – 31) | 0.173 |
| D-dimer (µg/ml) ^*^ | 1360 (735 – 2520) | 1820 (900 – 4192) | **0.033** |
| LDH (U/L) ^*^ | 296 (217 – 445) | 353 (253 – 560) | **0.001** |
| Serum ferritin (ng/ml) ^*^ | 478 (184 – 832) | 578 (307 – 1279) | **0.016** |
| NT-proBNP (pg/ml) ^*^ | 1016 (468 – 2896) | 2330 (1036 – 6544) | **<0.001** |
| hs-cTnT (ng/L) ^*^ | 29.4 (17.8– 50.0) | 74.3 (36.2 – 120.5) | **<0.001** |
| CRP (mg/dl) ^*^ | 4.61 (1.35 – 10.66) | 9.00 (3.90 – 15.62) | **0.001** |
| IL-6 (pg/ml) ^*^ | 31.8 (16.6 – 81.0) | 74.1 (30.9 – 120.6) | **0.002** |
| P/F | 298.0 ± 91.3 | 225.5 ± 84.9 | **<0.001** |

* The Mann-Whitney U test was used for the comparison between the two groups (no RASi during hospitalization vs RASi during hospitalization). Where not specified, unpaired two-tailed t-test was used for comparison of quantitative variables and χ² test was used for comparison of categorical variables.

Bold indicates signiﬁcance.

BMI: body mass index; GIC: geriatric index of comorbidity; CAD, coronary artery disease; HF: heart failure; AF: atrial fibrillation; TIA, transient ischemic attack; COPD: chronic obstructive pulmonary disease; BP: blood pressure; Hgb: haemoglobin; WBC: white blood cells; eGFR: estimated glomerular filtration rate; AST: aspartate aminotransferase; ALT: alanine aminotransferase; LDH: lactate dehydrogenase; NT-proBNP: N-terminal pro B-type natriuretic peptide; hs-cTnT: high-sensitive cardiac troponin T; CRP: C reactive protein; IL-6: interleukin-6; P/F: PaO2/FiO2 ratio (the ratio of arterial oxygen partial pressure in mmHg to fractional inspired oxygen expressed as a fraction, not a percentage).

Supplementary Figure S1. RASi therapy in the entire study population.


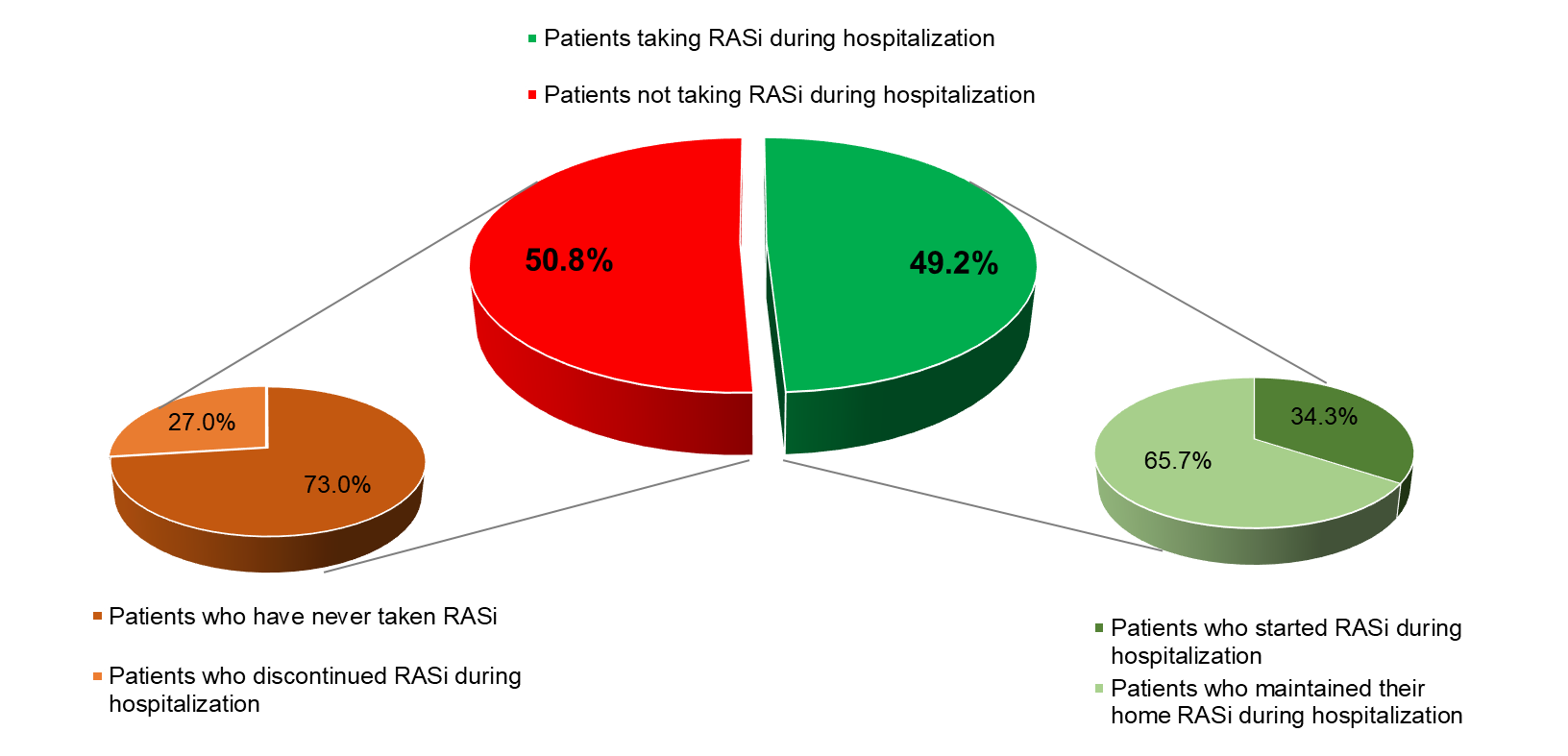


Supplementary Table S2. Cox regression analyses for in-hospital pneumonia-related mortality according to RASi therapy during hospitalization.

|  | HR | 95% CI | P |
| --- | --- | --- | --- |
| Model 1 | 0.58 | 0.40 – 0.83 | 0.003 |
| Model 2 | 0.58 | 0.41 – 0.84 | 0.004 |
| Model 3 | 0.52 | 0.30 – 0.89 | 0.016 |
| Model 4 | 0.54 | 0.34 – 0.84 | 0.007 |

CI: confidence interval.

Model 1: adjusted for age and sex.

Model 2: adjusted for age, sex and respiratory support.

Model 3: adjusted for age, sex, systolic blood pressure, ADL Hierarchy Scale, Ln(D-dimer), Ln(serum ferritin), Ln(high-sensitive cardiac troponin T).

Model 4: adjusted for age, sex, cognitive impairment, albumin, estimated glomerular filtration rate, Ln(C reactive protein), Ln(NT-proBNP).

Supplementary Table S3. Cox regression analyses for in-hospital mortality according to change in RASi therapy during hospitalization.

|  | Patients who started RASi during hospitalization (n° 57) vs patients who had never taken RASi (n° 133) | Patients who maintained their home RASi therapy (n° 109) vs patients who had never taken RASi (n° 133) | Patients who maintained their home RASi therapy (n° 109) vs patients who discontinued RASi during hospitalization (n° 38) |
| --- | --- | --- | --- |
|  | HR (95% CI) | HR (95% CI) | HR (95% CI) |
| Model 1 | 0.42 (0.24-0.73)* | 0.56 (0.37-0.84)* | 0.64 (0.37-1.11) |
| Model 2 | 0.43 (0.24-0.75)* | 0.56 (0.37-0.84)* | 0.66 (0.38-0.1.15) |
| Model 3 | 0.44 (0.22-0.90)* | 0.57 (0.33-0.1.01) | / |
| Model 4 | 0.39 (0.20-0.76)* | 0.62 (0.39-0.99)* | / |
| Model 5 | / | / | 0.55 (0.30-0.1.01) |

* p<0.05

CI: confidence interval.

Model 1: adjusted for age and sex.

Model 2: adjusted for age, sex and respiratory support.

Model 3: adjusted for age, sex, ADL Hierarchy Scale, Ln(D-dimer), Ln(serum ferritin), Ln(high-sensitive cardiac troponin T).

Model 4: adjusted for age, sex, cognitive impairment, albumin, estimated glomerular filtration rate, Ln(C reactive protein).

Model 5: adjusted for age, sex, cognitive impairment, Ln(C reactive protein).

Supplementary Table S4. Cox regression analyses for in-hospital mortality according to RASi therapy during hospitalization in patients with NT-proBNP ≥ 1800 pg/ml.

|  | HR | 95% CI | P |
| --- | --- | --- | --- |
| Model 1 | 0.45 | 0.27 – 0.76 | 0.003 |
| Model 2 | 0.50 | 0.30 – 0.85 | 0.011 |
| Model 3 | 0.37 | 0.19 – 0.75 | 0.006 |
| Model 4 | 0.49 | 0.27 – 0.88 | 0.018 |

CI: confidence interval.

Model 1: adjusted for age and sex.

Model 2: adjusted for age, sex and respiratory support.

Model 3: adjusted for age, sex, systolic blood pressure, ADL Hierarchy Scale, Ln(D-dimer), Ln(serum ferritin), Ln(high-sensitive cardiac troponin T).

Model 4: adjusted for age, sex, cognitive impairment, albumin, estimated glomerular filtration rate, Ln(C reactive protein).

Supplementary Table S5. General characteristics of the study population according to RASi therapy during hospitalization after propensity score matching.

| *Clinical parameters* | No RASi during hospitalization  (n° 58) | RASi  during hospitalization  (n° 58) | P |
| --- | --- | --- | --- |
| Age (years) | 87.6 ± 6.3 | 87.8 ± 5.5 | 0.858 |
| Sex (Female) | 63.8% | 70.7% | 0.429 |
| BMI (kg/m^2^) | 23.4 ± 3.6 | 24.9 ± 3.8 | 0.077 |
| ADL Hierarchy Scale: Assistance required | 24.1% | 29.3% | 0.741 |
| ADL Hierarchy Scale: Dependence | 48.3% | 48.3% |  |
| GIC (high comorbidity) | 70.9% | 73.2% | 0.787 |
| History of hypertension | 45.8% | 87.2% | <0.001 |
| History of CAD | 22.4% | 22.4% | 1.000 |
| History of HF | 32.8% | 31.0% | 0.842 |
| Type II Diabetes Mellitus | 25.9% | 20.7% | 0.510 |
| History of AF | 31.0% | 29.3% | 0.840 |
| Previous stroke/TIA | 15.5% | 17.2% | 0.802 |
| History of COPD | 12.7% | 24.6% | 0.109 |
| Cognitive impairment | 63.8% | 69.0% | 0.555 |
| Need for oxygen therapy | 56.9% | 56.9% | 0.651 |
| Need for ventilatory support | 32.8% | 27.6% |  |
| Systolic BP (mmHg) | 130.5 ± 17.9 | 133.1 ± 20.0 | 0.452 |
| Diastolic BP (mmHg) | 73.6 ± 12.0 | 74.8 ± 13.3 | 0.625 |
| *Laboratory parameters* |  |  |  |
| Hgb (g/dl) | 12.4 ± 2.2 | 12.8 ± 1.8 | 0.504 |
| WBC (n/mm^3^)^*^ | 7165 (4810 – 9955) | 6775 (4440 – 10610) | 0.832 |
| Neutrophils (n/mm^3^) ^*^ | 5270 (3328 – 9125) | 5065 (3508 – 8853) | 0.897 |
| Lymphocytes (n/mm^3^) ^*^ | 935 (608 – 1415) | 965 (635 – 1315) | 0.716 |
| eGFR (ml/min/1.73m^2^) | 54.8 ± 21.7 | 56.4 ± 22.4 | 0.705 |
| Albumin (g/dl) | 3.3 ± 0.5 | 3.3 ± 0.4 | 0.644 |
| AST (U/L) ^*^ | 29.5 (20.0 – 41.3) | 27.5 (21.0 – 40.5) | 0.512 |
| ALT (U/L) ^*^ | 20.5 (12.8 – 33.0) | 21.5 (11.3 – 31.3) | 0.729 |
| D-dimer (µg/ml) ^*^ | 1200 (800 – 3850) | 1890 (915 – 2790) | 0.524 |
| LDH (U/L) ^*^ | 298 (220 – 559) | 269 (225 – 378) | 0.604 |
| Serum ferritin (ng/ml) ^*^ | 558 (197 – 1002) | 535 (219 – 1003) | 0.747 |
| NT-proBNP (pg/ml) ^*^ | 1124 (545 – 2065) | 1351 (562 – 3475) | 0.405 |
| hs-cTnT (ng/L) ^*^ | 27.6 (17.9 – 56.0) | 38.7 (19.6 – 90.1) | 0.220 |
| CRP (mg/dl) ^*^ | 5.1 (2.2 – 14.2) | 3.7 (1.8 – 8.9) | 0.303 |
| IL-6 (pg/ml) ^*^ | 34.2 (15.7 – 99.8) | 55.1 (28.8 – 112.2) | 0.267 |
| P/F | 265.8 ± 89.0 | 250.6 ± 83.7 | 0.481 |

* The Mann-Whitney U test was used for the comparison between the two groups (no RASi during hospitalization vs RASi during hospitalization). Where not specified, unpaired two-tailed t-test was used for comparison of quantitative variables and χ² test was used for comparison of categorical variables.

RASi: renin-angiotensin-system inhibitors; BMI: body mass index; GIC: geriatric index of comorbidity; CAD, coronary artery disease; HF: heart failure; AF: atrial fibrillation; TIA, transient ischemic attack; COPD: chronic obstructive pulmonary disease; BP: blood pressure; Hgb: haemoglobin; WBC: white blood cells; eGFR: estimated glomerular filtration rate; AST: aspartate aminotransferase; ALT: alanine aminotransferase; LDH: lactate dehydrogenase; NT-proBNP: N-terminal pro B-type natriuretic peptide; hs-cTnT: high-sensitive cardiac troponin T; CRP: C reactive protein; IL-6: interleukin-6; P/F: PaO2/FiO2 ratio (the ratio of arterial oxygen partial pressure in mmHg to fractional inspired oxygen expressed as a fraction, not a percentage).
